# The impact of perceived algorithmic management on employees' physical and mental health: a chain mediation analysis of frustrated work autonomy and workplace AI replacement anxiety

**DOI:** 10.3389/fpubh.2026.1877221

**Published:** 2026-09-01

**Authors:** Zheng Ma, Xingzi Chen, Binhua Jing

**Affiliations:** 1 School of Economics and Management, Huainan Normal University, Huainan, China; 2 School of Accounting, Zhejiang University of Finance and Economics Dongfang College, Haining, China; 3 School of Business, Nanjing University, Nanjing, China

**Keywords:** algorithmic management, chain mediation analysis, employees' physical and mental health, frustrated work autonomy, perceived algorithmic management, workplace AI replacement anxiety

## Abstract

With the acceleration of digital transformation, perceived algorithmic management has gradually evolved into a widespread new type of occupational psychosocial hazard. From the perspective of occupational health, this study explores the negative association between perceived algorithmic management on employees' physical and mental health and systematically examines the chain mediating role of frustrated work autonomy and workplace AI replacement anxiety. Using a questionnaire survey method, this study collected data from 338 employees in multiple industries in China who were directly affected by perceived algorithmic management, SPSS 27.0 was used for preliminary statistical analyses (descriptive statistics, reliability testing, and correlation analysis), while AMOS 28.0 was used to construct and test the structural equation model (SEM) for confirmatory factor analysis and path estimation. Bootstrap resampling with 5,000 iterations was applied to assess indirect effects. The results show that perceived algorithmic management is significantly negatively associated with employees' physical and mental health; frustrated work autonomy and workplace AI replacement anxiety not only each play an independent mediating role in this relationship, but also constitute a significant chain mediating pathway. This study suggests a potential mechanism through which perceived algorithmic management in the digital era may be associated with occupational health risks, providing important empirical evidence for governments to formulate public health intervention policies, for enterprises to optimize management systems, and for the implementation of workplace mental health protection.

## 1 Introduction

Driven by the wave of artificial intelligence and digitalization, algorithms have deeply penetrated management systems across the global workforce, becoming a core mechanism for organizational coordination and information distribution. Perceived algorithmic management defined as employees subjective perceptions of algorithmic monitoring, performance evaluation, task allocation, and work constraints in their daily work (1, 2) now covers key processes such as task allocation, real-time monitoring, performance evaluation, and risk prediction, profoundly reshaping the daily logic of employees' work (3). This organizational transformation has not only altered traditional hierarchical leadership relationships, but has also brought unprecedented efficiency and transparency to the modern workplace, though the accompanying challenges have become increasingly apparent (4, 5). With the arrival of the Industry 5.0 era, patterns of human-machine interaction in the work environment have undergone fundamental changes. This digital transformation, as a new type of occupational exposure factor, has a profound associationon employees‘ mental health, job satisfaction, and overall wellbeing (6).

A recent synthesis of 39 empirical studies (7) identified algorithmic management as an emerging psychosocial risk in the workplace. As algorithmic management becomes more pervasive across sectors, its defining control mechanisms—frequent monitoring, automated appraisal, and fixed tasking (1, 2)—are showing stronger links to various occupational health issues. Employees who are persistently exposed to such algorithm-based systems often report heightened mental strain, emotional depletion, and burnout—all recognized as typical stress reactions in work settings (8, 9). In particular, algorithmic oversight tends to curb worker discretion while maintaining heavy performance demands (10). This combination has been tied to both poorer psychological wellbeing and elevated depressive symptoms (11). In addition, these management arrangements frequently reach beyond regular working hours via digital tools, which may cut into recovery time and worsen fatigue (12). Over time, the ongoing build-up of strain and tiredness—what the literature refers to as “digital mismatch”—could give rise to digital burnout, thereby undermining the long-term viability of the current workforce (13).

Safeguarding workers' physical and mental health has become a global consensus for achieving sustainable development. From the perspective of macro-level governance, improving occupational health management is not only a requirement for enterprises to fulfill their primary responsibilities, but also a core foundation for reducing public medical costs and maintaining social stability. Against this background, the Chinese government has also introduced a series of measures, such as the Healthy China 2030 Planning Outline and the Occupational Health Protection Action, aimed at enhancing the public's health management capacity by improving health-supportive environments. These policies reflect the global advocacy of healthy ways of working: employee health is no longer regarded merely as an operational cost, but as a core resource for an organization's long-term competitiveness (14). Upholding a people-centered management perspective can not only enhance employees' sense of organizational belonging, but also provide a micro-level foundation for improving the public health system (15). However, despite these policy efforts, the specific mechanisms through which perceived algorithmic management—as an emerging digital management practice—affects employee health remain insufficiently understood. This research gap motivates the present study.

Although the broad impact of perceived algorithmic management has attracted attention, the specific mechanisms through which it may be associated with employees‘physical and mental health still need to be further clarified. The standardization and metricization inherent in perceived algorithmic management, through intensive monitoring and data-driven evaluation, may obstruct the fulfillment of individuals' psychological needs, which could contribute to health impairments such as depression (3, 16). This data-driven trend in management may be linked to employees experiencing a profound sense of alienation and a decline in creativity (17). In addition, because algorithmic systems may physically or psychologically sever the traditional connection between employees and supervisors, individuals may be more likely to feel professionally isolated (18). Previous studies focusing on groups most deeply controlled by algorithms have also found that harsh performance surveillance and economic insecurity are associated not only with psychological stress responses, but may also be associated with physiological health risks such as skeletal disorders (19).

At present, although prior studies have documented a negative association between perceived algorithmic management and employee health outcomes (3, 8, 9), the sequential psychological mechanisms through which this relationship operates remain largely unexplored. Specifically, existing research has not examined whether perceived algorithmic management affects health through a stepwise process—first undermining work autonomy, then intensifying AI replacement anxiety, and ultimately impairing wellbeing. This gap is particularly significant in knowledge-intensive fields, where intelligent technologies often generate deep-seated “Workplace AI replacement anxiety”—a factor that has become a key concern for occupational wellbeing (20). This raises an important theoretical question: why should these two mediators operate in sequence rather than in parallel?

Theoretically, a serial mediation model is preferred over two separate mediators for two reasons. First, according to Conservation of Resources (COR) theory (21), resource loss triggers a cascade of further losses. When employees experience autonomy frustration—a loss of control over their work—they become more sensitive to external threats, such as the possibility of being replaced by AI. This logic suggests that autonomy frustration precedes and exacerbates AI replacement anxiety. Second, Self-Determination Theory (SDT) (22) posits that when the basic need for autonomy is thwarted, individuals become more vulnerable to external pressures and threats. In this context, the erosion of autonomy weakens employees' psychological resources, making them more likely to perceive AI as a personal threat. Thus, the sequence of autonomy frustration → AI replacement anxiety is theoretically grounded, whereas reversing the order—AI anxiety first, then autonomy frustration—would imply that external threat perceptions precede resource loss, which is less consistent with the loss spiral logic of COR and the need-thwarting mechanism of SDT. Building on this theoretical reasoning, the present study proposes a serial mediation model to test this sequential pathway.

To address this gap, the present study introduces frustrated work autonomy and workplace AI replacement anxiety as core mediating variables and constructs a serial mediation model. By investigating the sequential roles of these two variables, this study seeks to explore how perceived algorithmic management may be negatively associated with physical and mental health, thereby offering empirical findings that could inform occupational health interventions in digitally managed workplaces.

## 2 Literature review and hypotheses

### 2.1 Perceived algorithmic management and employees' physical and mental health

Despite its growing use as a management tool in the digital economy, researchers have yet to reach a shared understanding of what perceived algorithmic management entails. Most existing work has examined it within particular industry settings or specific occupational groups, yet three broad viewpoints can be identified.

Those adopting a control perspective view perceived algorithmic management largely as a system of technology-driven labor oversight. In contrast to conventional bureaucratic administration, it enables organizations to match workers with tasks through data collection across multiple points and standardized regulation of core job activities (10, 23). From this angle, algorithm-based management is fundamentally about exercising control: through continuous tracking and automated performance assessment, it shifts some managerial authority to technical systems, thereby altering how power and oversight are distributed within organizations.

A second perspective highlights the role of autonomous computing and intelligent inference as central to how algorithmic management operates. By drawing on large volumes of varied data to support automated choices, algorithmic systems are able to intervene in key managerial functions—a feature that sets them apart from older approaches (10, 24). This view draws attention to algorithms' capacity for independent decision-making, suggesting that they can offer both automated and advisory support, which may improve both efficiency and the precision of management choices.

A third view emphasizes the freeing potential of algorithmic management—its capacity to take over routine and repetitive duties, allowing employees to devote more time to creative tasks and enabling more productive human-machine collaboration. Supporters of this view argue that, with appropriate design, such systems can add to human skills rather than simply displacing them, potentially leading to more engaging and effective work environments.

The present study concentrates on the health implications of the control dimension of perceived algorithmic management—an aspect that, relative to the efficiency and enabling functions, has received comparatively limited empirical attention.

Taken together, these approaches describe perceived algorithmic management as drawing on deep learning and extensive data to combine internal and external information sources, reaching across different phases of organizational administration. Although it can improve operational efficiency, it has also been linked to notable shifts in workers' job conditions and overall health, making it a growing area of interest in organizational behavior and HRM research. Given their strong information-processing abilities and apparent objectivity, algorithmic systems can both inform managerial decisions and directly shape employees' work activities (25).

Building on these ideas, perceived algorithmic management can be understood as the set of technology-supported practices used in organizations; employees' subjective responses to, and evaluations of, these practices constitute their perceived algorithmic management. Operationally, we define it as workers' subjective perceptions of how intensely they are monitored by algorithms, how far algorithmic systems participate in decisions affecting them, and the extent to which algorithms constrain their work activities. While (26) examined algorithmic HR systems from an organizational design perspective, and (2) conceptualized algorithmic control through the “6Rs” framework (restricting, recommending, recording, rating, replacing, and rewarding), the present definition synthesizes these insights and adapts them to capture employees' subjective experiences of algorithmic management in their daily work. This operationalization thus reflects our own integration of existing conceptual work rather than a direct adoption of any single prior definition.

Earlier cross-sectional work has indicated that stronger algorithmic control is associated with greater work pressure and psychological strain, which in turn may be linked to poorer physical and mental health (8, 9). In the current study, we operationalize employees' physical and mental health as subjective psychological wellbeing, encompassing positive mood, vitality, and general life engagement, as measured by the WHO-5 WellBeing Index (27). More broadly, from an SDT standpoint, Gagné et al. (28) argued that algorithmic management can weaken workers' intrinsic motivation precisely because it frustrates their basic need for autonomy—a point that underpins the mediating pathway considered in the next section.

H1: Perceived algorithmic management has a significant negative predictive effect on employees' physical and mental health.

### 2.2 The mediating role of frustrated work autonomy

According to self-determination theory's core interpretation of individuals' need for autonomy (22, 29), frustrated work autonomy refers to the negative psychological experience in which individuals' core psychological need remains unmet and they lack a sense of control over their work because their right to autonomously arrange their work pace, choose their work methods, and control their work process is deprived by external forces, leaving them in a long-term state of rigid rule constraints and passive task execution. Frustrated work autonomy is one of the important sources of stress in the workplace and can directly disrupt an individual's psychological equilibrium, which may in turn relate to persistent psychological tension and emotional exhaustion (30). Research has found (31) that algorithm-centered digital management models, through the full chain of intelligent task allocation, real-time process monitoring, and rigid performance evaluation, exert refined and comprehensive deep control over employees' labor processes, greatly compressing employees' space for autonomous decision-making and flexible adjustment at work, thereby directly inducing frustrated work autonomy (32).

According to the core assumptions of Conservation of Resources theory (21), individuals have an inherent tendency to proactively preserve and acquire core resources necessary for survival and development, and the negative effects of resource loss are far greater than the positive effects of resource gain. As a core psychological resource that enables individuals to control their work, work autonomy, when continuously depleted without effective replenishment, may lead to strong stress responses and negative emotions, which in turn lead to a series of physical and psychological discomfort symptoms. Combined with the health impairment process in the Job Demands-Resources (JD-R) model (33, 34), perceived algorithmic management, due to its typical characteristics of strong control, high standardization, and low flexibility (10), falls within the category of high job demands. It continuously consumes individuals' psychological resources and work energy, accelerates the loss of work autonomy, and further amplifies the negative effects brought about by frustrated autonomy. Under the highly controlling environment of perceived algorithmic management, employees' space for autonomous decision-making is constantly compressed, and their psychological resources are continuously depleted, thereby intensifying frustrated work autonomy (32). The long-term imbalance and exhaustion of psychological resources can gradually cause individuals to fall into a state of burnout characterized by both physical and mental exhaustion (34), which not only gives rise to psychological problems such as low mood, anxiety, and irritability, but may be associated withphysiological problems such as sleep disorders and physical fatigue, ultimately damaging employees' physical and mental health. Based on the above theoretical reasoning, this study proposes the following hypothesis:

H2: Frustrated work autonomy mediates the relationship between perceived algorithmic management and employees' physical and mental health.

### 2.3 The mediating role of workplace AI replacement anxiety

The history of human technological development shows that every revolutionary technological innovation and transformation in modes of production has a structural impact on the workplace environment, labor processes, and employees' psychological states (35). From the perspective of the intrinsic link between technological development and employees' emotional responses, a large body of empirical research confirms that when emerging technologies become deeply embedded in daily work settings and reconstruct labor processes, they are associated with differentiated emotional feedback among employees (36). This emotional feedback is marked by a clear duality: on the one hand, technology can empower individuals, simplify repetitive labor, and improve work efficiency, thereby bringing positive emotional experiences and enhancing self-efficacy; on the other hand, technological change can also create uncertainty in career development, risks of job replacement, and a loss of control over work, which in turn may relate to negative emotional fluctuations among employees (17). Under the continuing impact of technology, these negative emotions may gradually evolve into complex psychological stress responses and physiological stress reactions, ultimately producing systematic negative effects on individuals' physical and mental health (37). This pattern is echoed in recent findings that AI-related job anxiety can reduce life satisfaction through psychological mechanisms (38), reinforcing the plausibility of a similar mediating process in algorithmic management contexts.

Workplace AI replacement anxiety is defined in this study as the negative emotional responses—including worry, unease, and fear—that employees experience when they perceive that their jobs or core professional skills may be substituted by artificial intelligence technologies. It is worth noting that Brougham and Haar (39) originally introduced the concept of STARA awareness (Smart Technology, Artificial Intelligence, Robotics, and Algorithms) to capture employees' general perceptions of technological change in the workplace, rather than providing a direct definition of AI replacement anxiety *per se*. The present definition draws on their broader conceptual framework while adapting it specifically to the context of algorithmic management, thereby reflecting our own operationalization of this construct rather than a direct adoption of their original wording.

As a core application of artificial intelligence in organizational contexts, perceived algorithmic management operates across multiple managerial functions through data-driven control, reshaping both management logic and labor processes (16). Prior research indicates that under sustained exposure to such technologies, employees tend to perceive that the skills they have developed over time and the tasks they routinely perform are gradually being supplanted by increasingly sophisticated AI systems, which in turn generates a pronounced sense of career insecurity and deep psychological distress (40). When such negative emotional states persist over time under high-stress conditions, they may progressively deplete individuals' psychological resources and disrupt their internal emotional balance, potentially contributing to a range of physical and psychological problems—including emotional exhaustion, heightened anxiety, irritability, and sleep disturbances (34, 41)—and ultimately may be associated with adverse effects on overall health and wellbeing. Based on the above reasoning, the following hypothesis is proposed:

H3: Workplace AI replacement anxiety plays a mediating role between perceived algorithmic management and employees' physical and mental health.

### 2.4 The chain mediating role of frustrated work autonomy and workplace AI replacement anxiety

Building on self-determination theory's classic explanation of individuals' intrinsic motivation, the need for autonomy is the fundamental source driving proactive behavior. In management contexts deeply permeated by algorithms, the core dimension first affected by the imbalance in adaptation between humans and intelligent systems is employees' work autonomy (22). When employees lose the power to make autonomous decisions over work pace and task execution methods, this further amplifies their perceived risk of AI-driven job replacement and intensifies their sense of career insecurity (40). This transmission logic aligns with the “technology–employee asymmetric adaptation perspective”: when algorithm-imposed job demands exceed an individual's capacity and fail to match employees' needs for autonomous development, employees can only passively conform to technological rules and find it difficult to adapt proactively to change.

Existing research shows that work autonomy and sense of control are key variables predicting technology-related job insecurity (40); meanwhile, perceived algorithmic management possesses the dual characteristics of rigid control and intelligent decision-making, making it a key driver of the compression of employees' autonomy space (2, 26). Sufficient work autonomy not only helps employees adapt more quickly to new technologies and processes, but also affects their work decision-making and degree of resource investment (42). Individuals with ample autonomy are more likely to satisfy their intrinsic need for competence, feel a stronger sense of control when facing algorithm-driven changes in work patterns, and maintain stable competence motivation with the support of psychological empowerment.

Theoretically, the ordering of autonomy frustration prior to AI replacement anxiety is grounded in two complementary frameworks. Conservation of Resources (COR) theory posits that resource loss triggers a cascade of further losses (21). When employees experience autonomy frustration, their remaining psychological resources become depleted, making them more vulnerable to external threats, including the possibility of being replaced by AI. Self-Determination Theory (SDT) further suggests that when the basic need for autonomy is thwarted, individuals become more sensitive to environmental pressures and threats (22). Thus, autonomy frustration is expected to precede and intensify AI replacement anxiety, whereas the reverse ordering would contradict the loss spiral logic of COR and the need-thwarting mechanism of SDT.

Combining the above theoretical reasoning and existing research findings, perceived algorithmic management mainly compresses employees' autonomous decision-making space and weakens individuals' control over their work through three core mechanisms—intelligent monitoring, automated decision-making, and data-driven feedback (1)—which may in turn contribute to frustrated work autonomy. The continued lack of work autonomy may be associated with reduced employees' ability and confidence to cope with technological change, may further amplifies their perception of AI-related occupational threat, and may intensifies workplace AI replacement anxiety. Prolonged exposure to replacement anxiety continuously depletes individuals' psychological resources and physiological energy, ultimately producing negative effects on physical and mental health (21).

It can thus be seen that perceived algorithmic management may indirectly and negatively affect employees' physical and mental health through the chain transmission path of “frustrated work autonomy → workplace AI replacement anxiety.” Accordingly, the following research hypothesis is proposed:

H4: Frustrated work autonomy and workplace AI replacement anxiety play a serial mediating role between perceived algorithmic management and employees' physical and mental health

**Figure 1:**
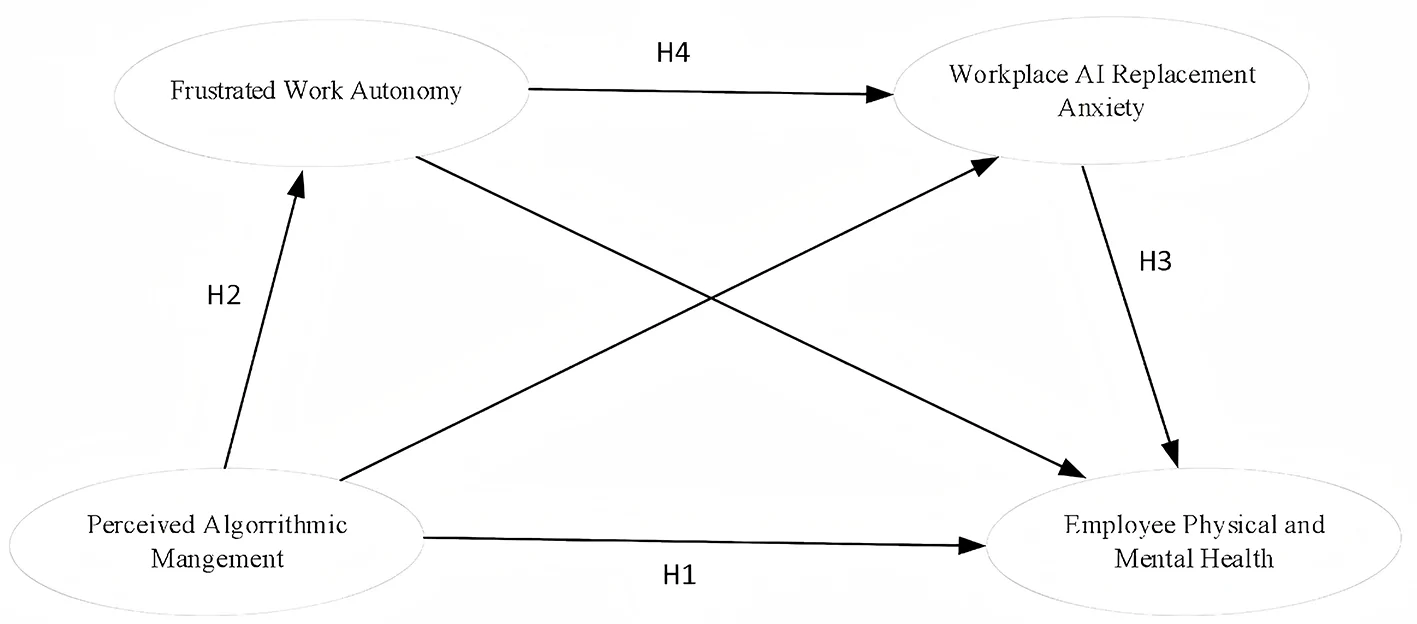
A conceptual model of how perceived algorithmic management affects employees' physical and mental health.

Based on the above literature review and theoretical deduction, the conceptual model of this study is constructed (see Figure 1).

## 3 Materials and methods

### 3.1 Variable measurement

This study sets four key variables around the core research question: the independent variable is perceived algorithmic management, the dependent variable is employees' physical and mental health, and frustrated work autonomy and workplace AI replacement anxiety are the chain mediating variables. To ensure the reliability and validity of the measurement results, all variables were measured using well-established scales that have been repeatedly validated in the fields of organizational behavior and management psychology; at the same time, in light of the actual scenarios of perceived algorithmic management in domestic workplaces and the characteristics of employee groups, the wording of the items was slightly localized to avoid measurement bias caused by contextual differences. Subsequent reliability and validity test results show that all scales in this study have good internal consistency reliability and structural validity, meeting the measurement requirements for empirical analysis.

#### 3.1.1 Perceived algorithmic management scale

The measurement of the independent variable, Perceived algorithmic management, was based on the perceived algorithmic management scale developed by Parent-Rocheleau et al. (1). The items were translated into Chinese by one researcher and then reviewed by a second researcher for accuracy. A third researcher back-translated the Chinese version into English to verify conceptual equivalence. After localization adjustments, 4 core items were retained, covering four core dimensions: algorithm-driven task allocation and scheduling, real-time data monitoring of the work process, algorithm-automated performance evaluation, and constrained autonomous decision-making space under algorithmic restrictions. A 7-point Likert scale was used, with 1 representing “strongly disagree” and 7 representing “strongly agree.” The mean score of the items was used as the final score, with higher scores indicating a greater intensity of perceived algorithmic management perceived by employees.

^*^The complete item wording (Chinese original and back-translated English versions) is provided in Supplementary Table S1.^*^

#### 3.1.2 Frustrated work autonomy scale

The measurement of the mediating variable, frustrated work autonomy, was based on the psychological need frustration scale developed by Chen et al. (43). In combination with the research context, 4 core items were selected, encompassing two dimensions: perceived involuntariness of work tasks and passive constraints on work behavior. A 7-point Likert scale was used, and the scoring rules were the same as above. Higher scores indicate more severe frustration of employees' work autonomy.

^*^The complete item wording (Chinese original and back-translated English versions) is provided in Supplementary Table S1.^*^

#### 3.1.3 Workplace AI replacement anxiety scale

The mediating variable, workplace AI replacement anxiety, was measured with reference to the perceived intelligent technology replacement scale developed by Brougham and Haar (39). Four core items were selected, covering anxiety perceptions across three dimensions: job replacement risk, the devaluation of professional skills, and restricted career development. A 7-point Likert scale was used, with scoring rules the same as above. Higher scores indicate higher levels of employees' workplace AI replacement anxiety.

^*^The complete item wording (Chinese original and back-translated English versions) is provided in Supplementary Table S1.^*^

#### 3.1.4 Employees' physical and mental health scale

The dependent variable—employees' physical and mental health—was assessed using the WHO-5 WellBeing Index, a measure systematically validated by Topp et al. (27). The original scale employs a 6-point Likert response format (0 = never, 5 = all the time). In the present study, a 7-point Likert scale (1 = strongly disagree, 7 = strongly agree) was adopted instead. This change was made primarily to keep the response format consistent with the other established scales used in the questionnaire, all of which also use a 7-point format, thereby reducing possible cognitive load on respondents caused by switching between different scale types.

We acknowledge that deviating from the original format requires a sound rationale. Fortunately, methodological evidence supports such an adjustment. Lozano et al. (44), using Monte Carlo simulations, found that the optimal number of response categories for Likert-type scales lies between four and seven. Fewer than four options tends to compromise reliability and validity, while more than seven yields only marginal psychometric improvement. A recent review by Taufique et al. (45) also concludes that “6–7 points maximize reliability and information” and that scales with fewer than five points may underperform. Accordingly, both the original 6-point and the modified 7-point format fall within the recommended optimal range, making the adaptation methodologically defensible.

To avoid any potential confusion arising from the format change, we have relabeled the construct as “Subjective Psychological Wellbeing (Modified WHO-5),” which more precisely reflects the content actually measured by the scale (i.e., positive mood, vitality, and general interest). In our sample, the modified version demonstrated satisfactory internal consistency, with a Cronbach's α of 0.829.

^*^The complete item wording (Chinese original and back-translated English versions) is provided in Supplementary Table S1.^*^

### 3.2 Sample data statistics

This study collected data through a questionnaire survey, with respondents mainly consisting of currently employed staff from various types of enterprises across multiple regions of the country. The survey was conducted online with the assistance of a professional online questionnaire platform (Wenjuanxing), and respondents were selected through a multi-stage sampling method to comprehensively collect questionnaire samples. Specifically, in the first stage, we identified five major industry sectors where algorithmic management is commonly applied: logistics and delivery, ride-hailing, e-commerce, manufacturing, and technology services. In the second stage, within each sector, we randomly selected companies that had implemented algorithmic management systems for at least one year. In the third stage, employees were randomly invited to participate via the platform's internal messaging system. No financial incentives were provided, but respondents were offered a summary of the research findings upon request. Invalid samples, including duplicate submissions by the same respondent and responses showing obvious patterned bias, were excluded based on indicators such as response completeness and IP duplication. Inclusion criteria were: (1) currently employed full-time in one of the target industries; (2) directly exposed to algorithmic management in daily work (defined as work tasks being assigned, monitored, or evaluated by algorithmic systems); and (3) having worked in the current position for at least three months. Exclusion criteria included: (1) part-time or temporary workers; (2) workers who had been in their current position for less than three months; and (3) incomplete questionnaires (missing more than 20% of items). To ensure meaningful exposure to algorithmic management, respondents were screened by a preliminary question: “To what extent are your daily work tasks assigned, monitored, or evaluated by algorithmic systems rather than human supervisors?” Only those who responded “to a moderate extent” or higher on a 5-point scale were included in the final sample. In the end, a total of 338 valid questionnaire samples were obtained. The target population was full-time employees in China who were directly exposed to algorithmic management. A total of 1,216 invitations were sent via the Wenjuanxing platform, and 338 valid responses were received (response rate = 27.8%). This rate is within the typical range for online surveys. The sample size of 338 is adequate for structural equation modeling, as samples of 200 to 400 are generally recommended for models with moderate complexity (46, 47).The descriptive statistical information is as follows:

Several procedural remedies were implemented during the survey design to minimize the risk of common method bias (54). First, respondents were assured of the anonymity and confidentiality of their responses to reduce social desirability bias. Second, the questionnaire items were arranged so that the independent variables (perceived algorithmic management) were presented before the dependent variable (physical and mental health), creating temporal separation. Third, different scale anchors were used for different measures to reduce response pattern consistency. Fourth, the survey instructions emphasized that there were no right or wrong answers, and respondents were encouraged to respond honestly.

In terms of gender distribution, males accounted for 50.9%, while females accounted for 49.1%, indicating a basically balanced gender ratio and effectively avoiding interference from gender bias in the research conclusions. In terms of age distribution, the sample covered all age groups, among which the 26–35 age group accounted for the highest proportion (34.9%), followed by the 36–45 age group (29.0%). Overall, the sample mainly consisted of young and middle-aged employees, which is consistent with the characteristics of the mainstream workforce in current perceived algorithmic management application scenarios. In terms of educational background, those with a bachelor's degree accounted for 50.9%, while the sample also covered junior college and below, master‘s, doctoral, and above levels of education, reflecting differences in employees' perceptions of perceived algorithmic management under different educational backgrounds. In terms of enterprise nature, the sample covered various types, including private/privately owned enterprises, state-owned enterprises, foreign-funded/joint ventures, and public institutions/government agencies, among which private/privately owned enterprises accounted for the highest proportion (34.0%), which is in line with the distribution characteristics of enterprise types where perceived algorithmic management is currently more widely applied. In terms of regional distribution, the sample covered eastern, central, western, and northeastern China, among which the eastern region accounted for the highest proportion (37.0%), followed by the central region (31.1%), while the western and northeastern regions accounted for 16.3% and 15.7%, respectively. This distribution feature is highly consistent with the current reality in which perceived algorithmic management is more widely applied in private enterprises and platform enterprises in economically developed regions, and can better reflect the real perceptions of employees in workplace environments where perceived algorithmic management is relatively prevalent. In terms of years of work experience, the sample covers the full range from less than 1 year to more than 15 years, among which the group with more than 15 years of work experience accounts for the highest proportion (30.2%), followed by the 9–15 years (inclusive) group (20.1%), reflecting differences in perceptions of perceived algorithmic management among employees with different workplace experience; in terms of position level, frontline employees (with no subordinates) account for 41.7%, and frontline managers account for 26.3%, while also covering middle-and senior-level managers, thus fully presenting the characteristics of workplace groups at different managerial levels; regarding job nature, the sample covers R&D, technical positions, production, manufacturing, logistics, marketing, sales, service positions, human resources, administration, finance, other functional positions, and other posts, among which R&D and technical positions account for the highest proportion (29.0%), which matches the widespread application of perceived algorithmic management in technical, production, and similar positions.

In summary, the survey sample in this study demonstrates a good balance of distribution and broad coverage in terms of gender, age, education level, enterprise type, region, years of work experience, position level, and job nature, and can effectively reflect the characteristic differences among employees from different backgrounds in their perceptions of perceived algorithmic management. It provides a reasonable sample basis for subsequent exploration of the mechanism linking perceptions of perceived algorithmic management and employees' physical and mental health, and thus has strong representativeness and typicality. The descriptive statistical information is presented in Table 1.

**Table 1:** Descriptive statistics of the survey sample.

Basic category	Option	Frequency	Percentage (%)
Gender	Male	172	50.9%
	Female	166	49.1%
Age	25 and under	35	10.4%
	26–35 years old	118	34.9%
	36–45 years old	98	29.0%
	46–55 years old	46	13.6%
	56 years old and above	41	12.1%
Education level	Junior college and below	48	14.2%
	Bachelor's degree	172	50.9%
	Master's degree	65	19.2%
	Doctorate and above	53	15.7%
Enterprise nature	State-owned enterprise	62	18.3%
	Private/Privately Owned Enterprises	115	34.0%
	Foreign-funded/Joint Venture Enterprises	68	20.1%
	Public institutions/government agencies	58	17.2%
	Others	35	10.4%
Area	Eastern Region	125	37.0%
	Central Region	105	31.1%
	Western Region	55	16.3%
	r	53	15.7%
Years of Work Experience	1 year or less	45	13.3%
	1–3 years (inclusive)	58	17.2%
	4–8 years (inclusive)	65	19.2%
	9–15 years (inclusive)	68	20.1%
	Over 15 years	102	30.2%
Job level	Frontline employee (no subordinates)	141	41.7%
	Frontline manager (e.g., supervisor, team leader)	89	26.3%
	Middle managers (e.g., department managers)	57	16.9%
	Senior managers (e.g., directors, vice presidents and above)	51	15.1%
Job Nature	R&D/Technical	98	29.0%
	Production/Manufacturing/Logistics	65	19.2%
	Marketing/Sales/Services	58	17.2%
	HR/Administration/Finance and Other Functional Roles	55	16.3%
	Other	62	18.3%

## 4 Results

To ensure the reliability of the study and the validity of related inferences, this study used SPSS 27.0 to conduct common method bias control, scale reliability testing, and basic variable analysis, and used Amos 28.0 to conduct scale validity and model fit testing. The core testing procedures included three major modules: common method bias testing, reliability analysis, and validity analysis, thereby eliminating random errors and systematic biases in the data and providing a solid and reliable data foundation for subsequent chain mediation effect testing and path analysis. Finally, the PROCESS macro program and the Bootstrap method were used to complete the chain mediation effect test.

By using the Harman single-factor test to control for common method bias, all scale items were included in exploratory factor analysis (EFA), and an unrotated principal component analysis was performed. The results showed that the variance explained by the first common factor was 24.382%, which is lower than the critical value of 30%, indicating that there is no serious common method bias in this study and that the issue of common source bias in the questionnaire data does not have a substantive impact on the research conclusions. We also ran a common latent factor (CLF) test in AMOS to check whether common method bias might distort our results. A method factor was added to the original measurement model, with all items loading on both their theoretical constructs and this factor. The model fit barely changed after adding the CLF—RMSEA stayed at 0.045, CFI moved from 0.968 to 0.973, and TLI from 0.961 to 0.962. The chi-square difference was significant (Δχ^2^ = 29.555, Δdf = 17, *p* = 0.0296), suggesting some method variance, but the fit indices were practically unchanged. We therefore concluded that common method bias does not seriously affect our findings as shown in Table 2.

**Table 2:** Fit indices comparison between baseline measurement model and CLF-controlled model.

Fit index	Criterion	Baseline model	CLF-controlled model	Change
CMIN	/	190.787	161.232	29.555
DF	/	113.000	96.000	17.000
CMIN/DF	< 3	1.688	1.679	0.009
RMSEA	< 0.05	0.045	0.045	0.000
RFI	>0.90	0.910	0.910	0.000
IFI	>0.90	0.968	0.973	−0.005
CFI	>0.90	0.968	0.973	−0.005
NFI	>0.90	0.925	0.937	−0.012
TLI	>0.90	0.961	0.962	−0.001

Since the core variables involved in this study were all measured using well-established scales, assessing the reliability of the measurement instruments is a core prerequisite for ensuring the effectiveness of subsequent empirical analysis. This study adopts the widely used Cronbach's α coefficient in academia as the core indicator for reliability testing. This coefficient is used to measure the homogeneity and internal consistency of the items within a scale, with values ranging from 0 to 1. The general criteria are as follows: if the α coefficient is greater than 0.8, the scale is considered to have good internal consistency reliability; if the coefficient falls between 0.7–0.8, reliability is considered acceptable; if the coefficient is less than 0.7, then the scale items need to be revised or deleted. The reliability test results of this study are shown in Table 3. The Cronbach's α coefficients for the four core variables—Perceived algorithmic management, frustrated work autonomy, workplace AI replacement anxiety, and employees' physical and mental health—were 0.811, 0.820, 0.865, and 0.829, respectively. The α coefficients of all variables were higher than the good reliability threshold of 0.8. These results indicate that all scales used in this study possess excellent internal consistency and structural stability, and that the measurement instruments are highly reliable, providing stable and precise quantitative data support for research on the relationship between Perceived algorithmic management and employees' physical and mental health.

**Table 3:** Questionnaire reliability analysis.

Variable	Cronbach's α	Number of items
Perceived algorithmic management	0.811	4
Frustrated work autonomy	0.820	4
Workplace AI replacement anxiety	0.865	4
Employees' physical and mental health	0.829	5

**Figure 2:**
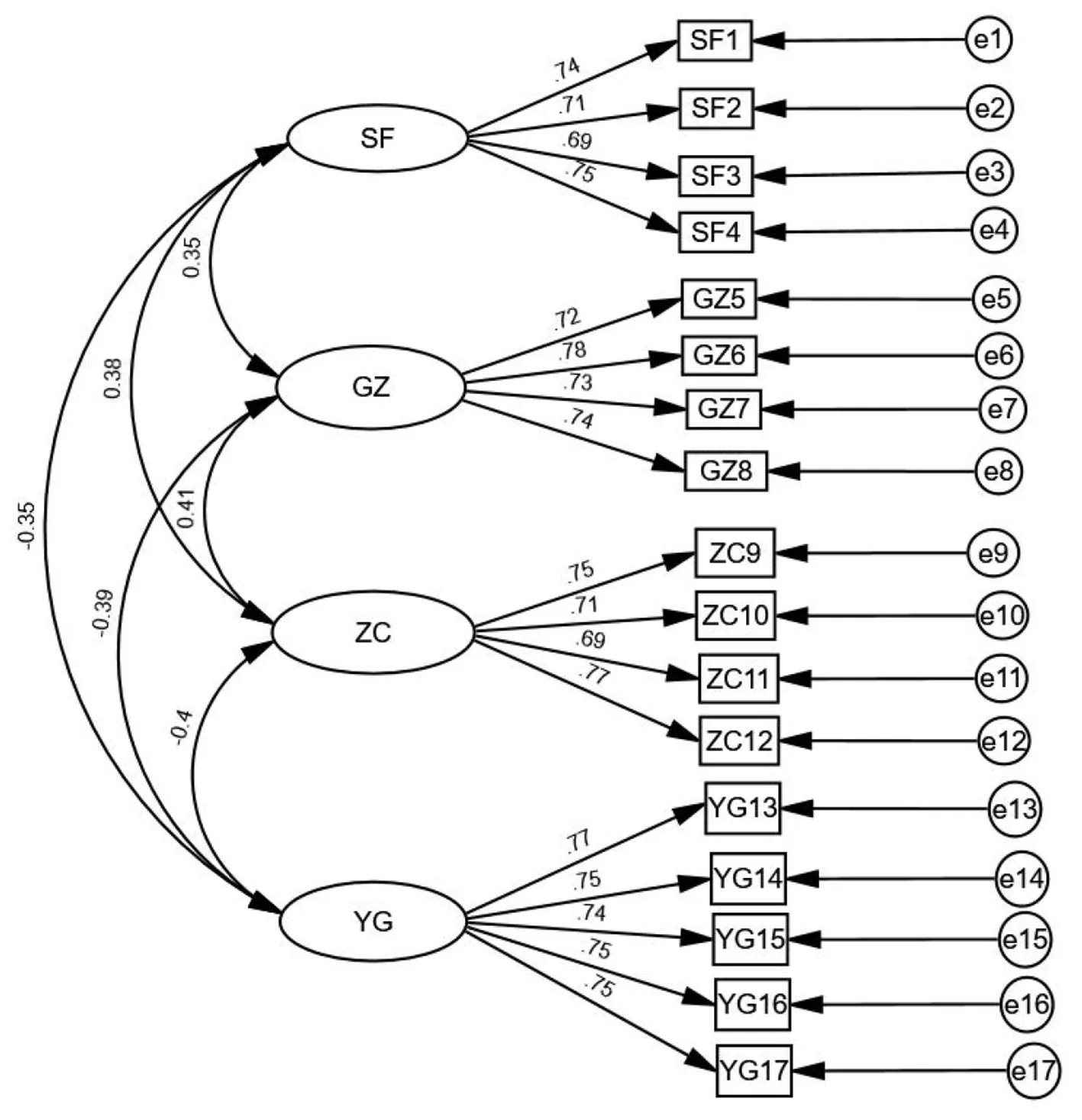
Confirmatory factor analysis (CFA) model.

To verify the structural validity of the scales, confirmatory factor analysis (CFA) was conducted using structural equation modeling. Because all scales were adopted from previously validated sources (1, 27, 39, 43), we proceeded directly to CFA without an initial exploratory factor analysis. The measurement model was specified as a first-order multifactor intercorrelation model for the four core variables. The analysis was performed using AMOS 28.0. The specific results are shown in Figure 2, providing a solid structural foundation for the accuracy of subsequent path analysis.

The specific experimental results are shown in Table 4. First, at the item observation level, the item loadings of all measured latent variables were relatively robust: “Perceived algorithmic management” had standardized loadings for each measurement item ranging from 0.689 to 0.748, “frustrated work autonomy” ranged from 0.723 to 0.776, ”workplace AI replacement anxiety“ ranged from 0.689 to 0.773, and “employees' physical and mental health” ranged from 0.741–0.772. All loading values not only clearly exceeded the statistical critical standard of 0.5, but also all reached an extremely significant level at *p* < 0.001, fully confirming the strong representational capacity of the observed items for the corresponding theoretical constructs.

**Table 4:** Results of confirmatory factor analysis.

Items	Variables	Standardized factor loading	AVE	CR	Unstandardized factor loading	S.E.	C.R.	P*P*
SF1	Perceived algorithmic management	0.737	**0.520**	**0.812**	1.001	0.060	16.777	
SF2		0.708			1.000			^***^
SF3		0.689			0.980			^***^
SF4		0.748			0.990			^***^
GZ5	Frustrated work autonomy	0.723	**0.553**	**0.821**	1.001	0.061	16.418	
GZ6		0.776			1.120			^***^
GZ7		0.727			1.010			^***^
GZ8		0.738			0.970			^***^
ZC9	Workplace AI replacement anxiety	0.751	**0.563**	**0.866**	1.002	0.057	17.547	
ZC10		0.707			0.900			^***^
ZC11		0.689			0.870			^***^
ZC12		0.773			1.040			^***^
YG13	Employees' physical and mental health	0.772	**0.550**	**0.830**	1.001	0.052	19.171	
YG14		0.746			0.990			^***^
YG15		0.741			0.970			^***^
YG16		0.747			0.950			^***^
YG17		0.745			0.950			^***^

^***^ indicates *p* < 0.001.

Bold values indicate the Average Variance Extracted (AVE) and Composite Reliability (CR) for each construct.

Secondly, in the assessment of convergent validity, by calculating composite reliability (CR) and average variance extracted (AVE), it can be seen that all core variables demonstrated excellent statistical indicators: ”Perceived algorithmic management“ (AVE = 0.520, CR = 0.812), ”frustrated work autonomy“ (AVE = 0.553, CR = 0.821), ”workplace AI replacement anxiety“ (AVE = 0.563, CR = 0.866), and ”employees' physical and mental health“ (AVE = 0.550, CR = 0.830) all had parameters that substantially exceeded the benchmark thresholds widely recognized in academia (AVE > 0.5, CR > 0.7). These results consistently indicate that the measurement scales used in this study possess excellent convergent validity, providing a highly reliable measurement basis for path analysis among the variables.

Discriminant validity is intended to confirm that there are statistically significant distinctions among the constructs of latent variables; that is, the correlation between a given latent variable and other variables should be significantly lower than the internal cohesion of its items. This study follows the classic Fornell-Larcker criterion to empirically test discriminant validity. The core assumption of this criterion is that the absolute value of the standardized correlation coefficient between any two constructs must be smaller than the arithmetic square root of the corresponding average variance extracted (AVE) (48).

This study first used Pearson correlation analysis to estimate the standardized correlation coefficients among the four core variables, and the results are shown in Table 5. The empirical findings reveal that the path relationships within the system exhibit a high degree of theoretical consistency: Perceived algorithmic management is significantly negatively correlated with employees' physical and mental health (*r* = −0.400, *p* < 0.001), and significantly positively correlated with frustrated work autonomy (*r* = 0.419, *p* < 0.001) and workplace AI replacement anxiety (*r* = 0.437, *p* < 0.001); frustrated work autonomy is significantly positively correlated with workplace AI replacement anxiety (*r* = 0.459, *p* < 0.001); workplace AI replacement anxiety is significantly negatively correlated with employees' physical and mental health (*r* = −0.424, *p* < 0.001). This close covariation lays a critical foundation for the subsequent path analysis of the chain mediating effect.

**Table 5:** Results of the discriminant validity test for each dimension.

Variable	Perceived algorithmic management	Frustrated work autonomy	Workplace AI replacement anxiety	Employees' physical and mental health
Perceived algorithmic management	**0.721**			
Frustrated work autonomy	0.419	**0.744**		
Workplace AI replacement anxiety	0.437	0.459	**0.750**	
Employees' physical and mental health	−0.400	−0.433	−0.424	**0.742**

Bold values on the diagonal represent the square root of the AVE for each construct, used to assess discriminant validity (Fornell?Larcker criterion).

Next, the square roots of the AVE values for each factor were calculated: Perceived algorithmic management was 0.721, frustrated work autonomy was 0.744, workplace AI replacement anxiety was 0.750, and employees' physical and mental health was 0.742. Comparative analysis shows that the absolute values of the standardized correlation coefficients between every pair of factors are all smaller than the square root of the corresponding dimension's AVE value. This statistical characteristic strongly confirms that the latent variables in this model possess excellent discriminant validity, rules out serious construct confusion bias, and verifies that each measurement scale has a high degree of independence and measurement precision in capturing specific research dimensions.

**Figure 3:**
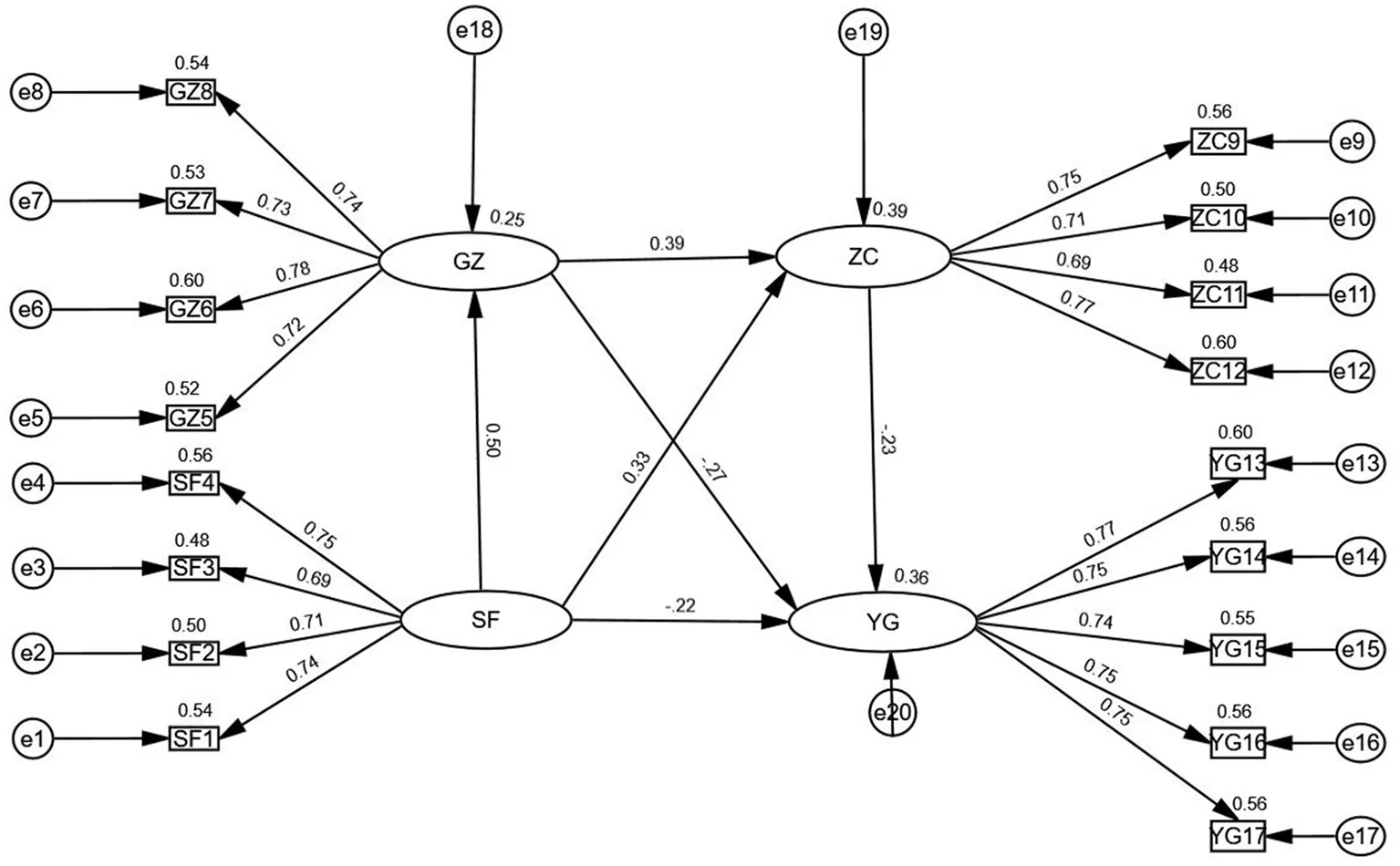
Standardized structural equation model.

Goodness-of-fit testing of the structural equation model (SEM) is a key step in quantitatively assessing the consistency between the theoretically hypothesized model and the covariance matrix of the sample data. It not only determines the statistical validity of the internal path coefficients of the model, but also serves as the foundation for the scientific soundness of the entire chain mediation model. Drawing on the goodness-of-fit indicator system commonly used in research in organizational behavior and psychology, this study selected the chi-square/degrees of freedom ratio, absolute fit indices, and multiple incremental fit indices to form a comprehensive evaluation system for testing the adaptability of the constructed theoretical model. According to the conceptual model hypothesized in this study, through the fitting and revision of the questionnaire data, the various path relationships were ultimately established, and the revised standardized model paths are shown in Figure 3.

The specific results are shown in Table 6, indicating that all key indicators reflecting model fit have reached ideal standards: the chi-square/degrees of freedom ratio CMIN/DF is 1.688, which falls within the excellent range of 1–3, indicating that the model structure is concise and has strong explanatory power for the data; the absolute fit index RMSEA is 0.045, significantly lower than the critical threshold of 0.05, showing that the model has minimal approximation error; the incremental fit indices RFI, IFI, CFI, NFI, TLI are 0.910, 0.968, 0.968, 0.925, and 0.961, respectively, all exceeding the criterion of 0.9. This indicates that the chain mediation model constructed in this study fits the actual survey data extremely well and provides a scientific basis for proceeding to subsequent path estimation and hypothesis testing.

**Table 6:** Model fit.

Indicator	CMIN	DF	CMIN/DF	RMSEA	RFI	IFI	CFI	NFI	TLI
Acceptable range			< 3	< 0.05	>0.9	>0.9	>0.9	>0.9	>0.9
Measured Values	190.787	113.00	1.688	0.045	0.910	0.968	0.968	0.925	0.961

On the premise of a good model fit, this study used the path coefficients in the structural equation model to examine the direct effect of Perceived algorithmic management on employees' physical and mental health, as well as the mediating path effects of frustrated work autonomy and workplace AI replacement anxiety between the two. Path coefficients directly reflect the strength of the influence relationship between variables, and the critical ratio (C.R.) is used to test the significance of the path coefficients. All demographic variables—gender, age, education, enterprise type, region, years of work experience, job level, and job nature—were entered as covariates in the structural equation model, regressed on all endogenous variables. None of these control variables had a significant effect (all *p* > 0.05), and the significance and direction of all hypothesized paths remained unchanged after controlling for these variables. The specific results are shown in Table 7.

**Table 7:** Structural equation model path coefficients.

Path	Unstandardized coefficient	S.E.	C.R.	*P*	Standardized coefficient
Perceived algorithmic management → Frustrated work autonomy	0.517	0.073	7.073	^***^	0.505
Frustrated Work Autonomy → Workplace AI replacement anxiety	0.412	0.078	5.263	^***^	0.391
Perceived algorithmic management → Workplace AI replacement anxiety	0.355	0.079	4.491	^***^	0.329
Workplace AI replacement anxiety → employees' physical and mental health	−0.241	0.081	−2.990	0.003	−0.234
Perceived algorithmic management → employees' physical and mental health	−0.240	0.083	−2.895	0.004	−0.216
Frustrated work autonomy → employees' physical and mental health	−0.293	0.084	−3.512	^***^	−0.270

^***^ indicates *p* < 0.001.

This study first examined the direct effect of Perceived algorithmic management on employees' physical and mental health. The results showed that the unstandardized path coefficient from Perceived algorithmic management to employees' physical and mental health was −0.240, with a significance level of *p* < 0.01. The path coefficient was significantly negative, and hypothesis H1 was supported: the higher the intensity of perceived algorithmic management perceived by employees, the lower their level of physical and mental health.Thus, the null hypothesis—that perceived algorithmic management has no significant effect on employees' physical and mental health—was rejected.

The test results for the path ”perceived algorithmic physical and mental health” showed that Perceived algorithmic management had a significant positive predictive effect on frustrated work autonomy, with an unstandardized path coefficient of 0.517 and *p* < 0.001, while the effect of frustrated work autonomy on employees' physical and mental health also reached a significant level, with an unstandardized path coefficient of −0.293. Hypothesis H2 was supported. Thus, the null hypothesis of no mediating effect was rejected.

According to the path logic of research hypothesis H3, the unstandardized path coefficient from Perceived algorithmic management to workplace AI replacement anxiety is 0.355, with a C.R. value of 4.491 (greater than 3.29), and *p* < 0.001. The path coefficient is significantly positive, indicating that Perceived algorithmic management significantly increases individuals' level of workplace AI replacement anxiety; the unstandardized path coefficient from workplace AI replacement anxiety to employees' physical and mental health is −0.241, indicating that an increase in workplace AI replacement anxiety significantly harms employees' physical and mental health, thus supporting hypothesis H3. Thus, the null hypothesis of no mediating effect was rejected.

To explore the stepwise influence pattern among variables, this study focused on testing the chained mediation path proposed in Hypothesis H4: Perceived algorithmic management → frustrated work autonomy → workplace AI replacement anxiety → employees' physical and mental health. As shown in Table 7, the path coefficient of Perceived algorithmic management → frustrated work autonomy was 0.517 (*p* < 0.001), and the path coefficient of workplace AI replacement anxiety → employees' physical and mental health was −0.241 (*p* < 0.01). The key sub-path of frustrated work autonomy → workplace AI replacement anxiety yielded a path coefficient of 0.412, with a significance level of *p* < 0.001, indicating that frustrated work autonomy significantly exacerbates workplace AI replacement anxiety, thus supporting Hypothesis H4. Thus, the null hypothesis of no serial mediation was rejected.

To further verify the robustness of the path coefficients, this study used the Bootstrap resampling method proposed by Preacher and Hayes (49) to conduct a final test of the model's mediating effects. This method does not require the assumption that the data follow a normal distribution, and the confidence intervals it constructs are more precise and have greater statistical power, making it the gold standard for testing mediating effects. In this test, the number of resamples was set to 5,000. If the 95% confidence interval for a given path does not include 0, it indicates that the effect of that path is significant. The specific test results for the mediating effects are shown in Table 8: the mediating effect value of the “Perceived algorithmic management—frustrated work autonomy—employees' physical and mental health” path was −0.154, with a Bootstrap standard error of 0.067 and a 95% confidence interval of [−0.318, −0.050], which does not include 0, indicating that the independent mediating effect of frustrated work autonomy was significant and further confirming Hypothesis H2; the mediating effect value of the “Perceived algorithmic management—workplace AI replacement anxiety—employees' physical and mental health” path was −0.087, with a Bootstrap standard error of 0.049 and a 95% confidence interval of [−0.226, −0.016], excluding 0, indicating that the independent mediating effect of workplace AI replacement anxiety is significant, further confirming hypothesis H3; the mediating effect value of the chain path “ Perceived algorithmic management—frustrated work autonomy—workplace AI replacement anxiety—employees' physical and mental health” is −0.052, with a Bootstrap standard error of 0.027, and a 95% confidence interval of [−0.128, −0.012], which does not include 0, once again confirming that hypothesis H4 is supported.

**Table 8:** Results of the mediation effect test.

Path	Mediation effect value	BootSE	BootLLCI	BootULCI
Perceived algorithmic management—Frustrated work autonomy—Employees' physical and mental health	−0.154	0.067	−0.318	−0.050
Perceived algorithmic management—Workplace AI replacement anxiety—Employees' physical and mental health	−0.087	0.049	−0.226	−0.016
Perceived algorithmic management—Frustrated work autonomy—Workplace AI replacement Anxiety—Employees' physical and mental health	−0.052	0.027	−0.128	−0.012

To see whether our proposed serial mediation model (perceived algorithmic management → frustrated work autonomy → workplace AI replacement anxiety → health) was the best option, we compared it against two alternatives. The first alternative reversed the order of the two mediators (perceived algorithmic management → workplace AI replacement anxiety → frustrated work autonomy → health). The second treated the two mediators as parallel rather than sequential (perceived algorithmic management → both mediators → health).

As shown in Table 9, the reversed-order model gave exactly the same fit as our proposed model (CMIN/DF = 1.688, RMSEA = 0.045, CFI = 0.968). This is not surprising—when two mediators are strongly correlated, reversing their order often produces an equivalent model. What matters is theoretical grounding: COR theory suggests that resource loss (autonomy frustration) comes first and makes people more sensitive to external threats (AI anxiety), whereas the reverse order would put the threat before the loss. The parallel model, however, fit noticeably worse (CMIN/DF = 1.937, RMSEA = 0.053, RFI = 0.897), with RMSEA above 0.05 and RFI below 0.90. So while the data cannot distinguish between our order and the reversed order statistically, the theoretical logic clearly favors our sequence, and the parallel model is clearly inferior.

**Table 9:** Fit indices for competing models.

Model	CMIN	DF	CMIN/DF	RMSEA	RFI	IFI	CFI	NFI	TLI
Acceptable range	/	/	< 3	< 0.05	>0.9	>0.9	>0.9	>0.9	>0.9
Proposed serial mediation model	190.787	113.00	1.688	0.045	0.910	0.968	0.968	0.925	0.961
Competing Model 1 (reversed order)	190.787	113.00	1.688	0.045	0.910	0.968	0.968	0.925	0.961
Competing Model 2 (parallel mediation)	220.866	114	1.937	0.053	0.897	0.956	0.956	0.913	0.947

## 5 Discussion

### 5.1 Theoretical contributions

This study mainly draws on two mature theoretical frameworks—Conservation of Resources theory and Self-Determination Theory—to systematically verify the damaging mechanisms and transmission pathways through which Perceived algorithmic management in digital work environments affects the mental health and occupational wellbeing of formally employed workers. At present, attention in the fields of public health and occupational epidemiology to digital labor management has largely been limited to gig workers in platform economies such as food delivery and ride-hailing, with a primary focus on emotional exhaustion and physical health effects caused by instability and high-intensity labor, as reflected in the empirical discussion and intervention appeal by Freni-Sterrantino and Salerno in this journal regarding the health effects of the gig economy (50). However, as algorithmic systems increasingly penetrate the entire process of traditional organizations, there is still a lack of systematic unpacking and causal mechanism analysis regarding the long-term health toll that this form of digital control may impose on ordinary employees working under formal contracts. In response to this research gap, this study introduces frustrated work autonomy and workplace AI replacement anxiety as core psychological mechanism variables, fully reconstructing the pathological psychological process through which employees move from exposure to digital stressors to eventual physical and mental depletion. This study not only fills a research gap at the intersection of digital labor and occupational health, but also provides a solid theoretical reference for organizations seeking to implement effective workplace psychological interventions during digital transformation. The specific theoretical contributions are as follows.

#### 5.1.1 Revealing the micro-level psychological pathogenic mechanisms of digital health risks and completing the exposure-health explanatory chain

Previous macro-level studies on perceived algorithmic management have mostly focused on system design or labor relations, often treating employees as passive recipients and rarely opening the “black box” of how algorithmic systems may be associated withindividuals' physical and mental wellbeing through internal mechanisms. This study lowers the level of analysis to employees' micro-level cognition and emotions, and empirically confirms that perceived algorithmic management, as a new type of occupational psychosocial hazard, exerts its negative effects essentially by depriving employees of their basic psychological need for autonomy and control. This finding not only supplements algorithmic control theory with micro-level empirical evidence from health psychology, but also clarifies the dynamic stress response process employees undergo when exposed to digital stressors.

#### 5.1.2 Bridging cross-disciplinary research on perceived algorithmic management and occupational health, and clarifying the chained transmission mechanism of multiple psychological depletions

Existing occupational health research has largely focused on traditional work stressors, while lacking a systematic examination of emerging digital burdens; at the same time, research on perceived algorithmic management has mostly concentrated on objective outcomes such as organizational performance, overlooking the potential health costs. By introducing two mediating variables, this study not only verifies the independent mediating roles of frustrated work autonomy and workplace AI replacement anxiety, but also provides the first empirical evidence for the chain transmission path of Perceived algorithmic management—frustrated work autonomy—workplace AI replacement anxiety—psychophysical health depletion. This effectively clarifies how process-oriented control pressure and future-oriented career anxiety jointly deplete individuals' psychological buffering resources, constructing a complete logical loop from external digital exposure to the deterioration of internal psychological states and ultimately to impaired public health outcomes.

#### 5.1.3 Redefining workplace AI replacement anxiety as a specific occupational mental health risk and broadening the boundaries of intervention in the digital-intelligence era

Existing literature has mostly treated workplace AI replacement anxiety as a generalized social emotion, and has rarely examined its mediating destructive effect on occupational health within specific management contexts. As confirmed by the latest research of Li and Pang (13), the digital mismatch between the rigid control of technological systems and individuals' actual needs is a core driver behind the sharp rise in stress and digital burnout among groups in the modern workplace (13). This study explicitly defines workplace AI replacement anxiety as a persistent state of psychological stress experienced by employees under the pressure of algorithmic environments due to threats to their core values. This shift in perspective not only enriches occupational psychology theory in the AI era, but also provides an entirely new entry point for preventing and precisely intervening in the collective “digital burnout” that may be associated withdigital technologies.

#### 5.1.4 Expanding the coverage of digital health monitoring and filling the empirical gap in traditional formally employed groups

Current literature examining the negative health effects of algorithms has been seriously limited in its samples to non-standard workers in the on-demand economy. However, this study focuses on full-time employees in traditional firms and provides evidence consistent with the view thatthe health-depleting effects of perceived algorithmic management are also widely pervasive in formal bureaucratic organizations. This conclusion substantially extends the theoretical boundary of the health hazards of algorithms from the platform economy to routine work settings across all industries. It suggests to public health scholars and policymakers that the occupational health risks brought about by digital management have become highly systemic and widespread, and that the vast population of formal employees must be incorporated into national occupational health monitoring networks and protection systems in the digital age.

### 5.2 Practical implications

Based on the empirical findings of this study—that Perceived algorithmic management can significantly harm employees' physical and mental health by weakening their work autonomy and heightenedworkplace AI replacement anxiety—this study argues that perceived algorithmic management brings not only organizational management challenges, but also a public health and occupational health issue that urgently needs to be addressed in the digital age. Drawing on current intervention concepts in occupational epidemiology and workplace health promotion, this study proposes the following practical intervention recommendations aimed at preventing digital occupational health risks from three dimensions: environmental control (primary prevention), monitoring intervention (secondary prevention), as well as policy governance and individual empowerment.

#### 5.2.1 Primary prevention in the workplace: reshaping the logic of algorithm design to control occupational psychosocial hazards at the source

This study confirms that frustrated work autonomy is the primary pathogenic pathway through which perceived algorithmic management harms health. From a public health perspective, the most effective way to prevent occupational diseases is to eliminate hazards at the source. Therefore, when designing and deploying algorithmic systems, companies must implement the principle of Health in All Policies and incorporate safeguards for employee autonomy into the underlying framework of the system. This requires algorithmic applications to shift from purely automated, one-way control to two-way human-machine collaborative empowerment. For example, companies should reserve for employees the necessary discretion to make adjustments in algorithmic scheduling and task allocation, so as to avoid excessively dense, dehumanizing instructions that may contribute towork overload (2). By optimizing job design to give employees a sense of control, psychological stress and physiological exhaustion induced by frustrated autonomy can be blocked at the root (3).

#### 5.2.2 Occupational health interventions: promote digital hazard communication and skills empowerment to cut off the transmission chain of AI replacement anxiety

The results of this study show that workplace AI replacement anxiety is a key mediating bridge causing health impairment. In occupational health management, eliminating fear caused by information asymmetry is one of the core intervention strategies. Companies should proactively break open the algorithmic “black box” and establish standardized digital hazard communication mechanisms to transparently disclose to employees the operating logic of algorithms, the basis for performance evaluation, and decision-making standards, thereby reducing job insecurity caused by the unknown (36). In addition, organizations should regularly provide targeted digital skills training and career transition counseling to help workers correctly understand the supportive-tool nature of algorithms, transforming their generalized fear of being replaced by machines into core literacy in mastering digital-intelligent tools, thereby effectively cutting off the pathological transmission chain of anxiety (39).

#### 5.2.3 Workplace Health Promotion (WHP): establish a normalized system for mental health screening and secondary prevention

From the long-term perspective of public health, employees' physical and mental health is a core asset for sustaining social productivity and the sustainable development of organizations (14). In the face of the hidden and persistent pressure of perceived algorithmic management, enterprises should embed mental health protection into daily operations and establish normalized mechanisms for occupational health monitoring and screening. Through regular psychological assessments, wearable device monitoring, or employee assistance programs, organizations can identify at an early stage those at high risk of emotional exhaustion and suboptimal health under intense algorithmic pressure, and provide timely psychological first aid and counseling interventions (15). Building on Self-Determination Theory's emphasis on basic psychological needs (22) and Conservation of Resources theory's focus on protecting core resources (21), safeguarding employees' health and safety is essential for maintaining their motivation, wellbeing, and long-term productivity. Implementing people-centered health management, balancing technological efficiency with humanistic care, may be one way to reduce the risk of collective digital burnout.

#### 5.2.4 Public health policy governance: incorporating digital labor protection into the national occupational safety and health regulatory framework

As perceived algorithmic management continues to spread comprehensively across traditional industries, the collective health risks it brings may extend beyond the capacity of individual enterprises to manage, suggesting a possible role for government macro-level public health policy (10). It may be worth considering whether relevant authorities, such as national health commissions and labor inspectorates, could take steps to improve occupational safety and health standards for the digital-intelligent era and clarify the health red lines for the application of algorithms in human resource management. For example, drawing on advanced international labor protection legislation, policies and regulations could be introduced to prohibit algorithms from conducting excessive physiological monitoring, grant employees the right to appeal automated decisions, and guarantee the right to disconnect (17, 18). At the same time, industry associations could also play a part in formulating industry evaluation standards for healthy algorithms and, through institutional safeguards, contribute to a broader public health framework for the digital workforce.

#### 5.2.5 Health education at the individual level: enhancing workers' digital health literacy and psychological resilience

According to the conservation of resources theory, when individuals are exposed to environmental stress, they will actively mobilize internal resources to buffer health loss (21). Beyond macro-level policies and corporate interventions, workers' own health literacy is also an important line of defense against digital hazards. Public health education should guide workers to proactively improve their digital health literacy, enabling them not only to master the hard operational skills needed to cope with perceived algorithmic management, but also to learn how to draw psychological boundaries between work and life in high-pressure digital environments (39). Employees should learn to recognize their own emotional and stress signals, and build psychological capital (such as resilience) through positive stress management techniques and seeking social support, thereby establishing a psychological buffer of self-protection when facing the potential harms of perceived algorithmic management (22, 51).

In conclusion, perceived algorithmic management is not only a product of technological progress, but also a key variable reshaping the determinants of modern occupational health. In the face of the public health risks that may be associated with it, multidimensional and coordinated interventions by governments, enterprises, and individuals may be worth exploring to guide technology use in a healthier direction, so that digital transformation truly serves comprehensive human wellbeing and the sustainable development of society.

## 6 Conclusions

With Perceived algorithmic management as the core occupational exposure factor, this study systematically explored the mechanisms through which the digital work environment affects employees' physical and mental health by constructing a chain mediation model, and clarified the transmission path of frustrated work autonomy and workplace AI replacement anxiety in the workplace within this process. The findings reveal how an algorithm-driven management model, as a new type of occupational psychological stressor, impairs employees' physical and mental wellbeing by depriving individuals of psychological resources. This finding is highly consistent with recent research on interventions for digital workplace health, namely that algorithmic monitoring and automated decision-making have become occupational health risk factors that must be confronted in modern public health (52). Based on the empirical analysis, this study draws the following four core conclusions.

### 6.1 Perceived algorithmic management constitutes a significant digital health risk and has a significant negative predictive effect on physical and mental health

The study confirms that Perceived algorithmic management has a direct negative impact on employees' physical and mental health, further supporting the view that perceived algorithmic management, as a new form of digital burden, undermines occupational health (9, 20). From a public health perspective, this management model is not merely a technological tool, but also a round-the-clock source of occupational stress exposure. Through high-frequency task allocation and real-time performance monitoring, it induces individuals' psychological stress responses and physiological exhaustion (2). When employees perceive that their work processes are being highly quantified and controlled by algorithms, the lack of human-centered support mechanisms is associated with a systematic decline in positive health indicators such as emotional wellbeing, energy levels, and inner calm (27). The conclusions of this study indicate that the negative health effects of perceived algorithmic management are not limited to gig workers in the platform economy, but also manifest as significant health depletion in formal employment settings within traditional enterprises.

### 6.2 Frustrated work autonomy is a key psychological mechanism through which perceived algorithmic management harms occupational health

Empirical results show that frustrated work autonomy plays a significant mediating role between Perceived algorithmic management and physical and mental health. Based on self-determination theory, autonomy is one of the three core needs for maintaining occupational mental health. Research has found that the rigid constraints of perceived algorithmic management directly sever individuals' control over work pace and decision-making. This sense of deprivation is associated with the persistent frustration of basic psychological needs, which in turn may be related toa series of anxiety and depression symptoms (4, 27). When algorithms automatically assign tasks and compress the space for autonomous decision-making, workers experience a strong sense of passivity and oppression (43). This long-term state of psychological imbalance mayaccelerates the depletion of psychological resources and ultimately is associated with a deterioration in their overall health.

### 6.3 Workplace AI replacement anxiety acts as a mediating bridge between digital stress and impaired health

Research has found that workplace AI replacement anxiety is likewise an important mediating pathway through which perceived algorithmic management affects physical and mental health. As algorithmic systems expand their coverage of job positions, employees' fear of technological unemployment has evolved into a widespread occupational psychological disorder, and this uncertainty greatly increases employees' psychological burden and reduced their occupational wellbeing (20). This anxiety, rooted in long-term career expectations, can may be associated withsustained stress responses, which in turn may relate to typical subhealth manifestations such as reduced energy and depressed mood (27). This indicates that perceived algorithmic management brings not only immediate task pressure but also long-term mental health risks to the public health system by reshaping individuals' perceptions of career security.

### 6.4 Frustrated work autonomy—workplace AI replacement anxiety constitutes a chain transmission pathway that harms employee health

This study's most central contribution lies in revealing the chained process through which Perceived algorithmic management—frustrated work autonomy—workplace AI replacement anxiety—physical and mental health depletion operate. This finding constructs a complete pathological chain from external occupational environmental pressure to the depletion of internal psychological resources, then to long-term career anxiety, and ultimately to health damage (10). The logic is that excessive algorithmic control first deprives employees of their sense of autonomous control (1), which may in turn relate toexperience work frustration; this sense of losing control further intensifies individuals' negative evaluation of the risk of technological replacement, making them feel deeply that their value can be quantified and replaced by algorithms (39); ultimately, the accumulation of this compound psychological pressure is associated with a significant collapse in physical and mental health (27). This conclusion echoes the core logic in occupational psychology that “the loss of control intensifies uncertainty” and provides a systematic explanatory framework for understanding the process of occupational health deterioration in the digital-intelligent era.

## 7 Research limitations and prospects

This study, while systematically examining the pathological transmission mechanism through which Perceived algorithmic management affects employees' physical and mental health, still has certain limitations. First, constrained by the cross-sectional research design and a single self-report questionnaire, this study cannot firmly establish the strict causal relationships among variables and cannot completely avoid common method bias (2, 26, 53). Moreover, the cross-sectional design of this study also precludes testing the causal ordering of the proposed mediation chain. Although we have grounded the sequence of “autonomy frustration → AI replacement anxiety” in theoretical reasoning (see Section 2.4), we cannot empirically rule out alternative orderings, such as AI anxiety preceding autonomy frustration, or parallel mediation.Future research should consider incorporating multi-source objective data and longitudinal tracking designs to accurately capture the long-term dynamic effects of digital stress exposure on employee health. Second, this study treated Perceived algorithmic management as a single overall construct and failed to deeply analyze the differentiated impacts that different dimensions, such as real-time monitoring and automated performance evaluation, may have on occupational health; subsequent research urgently needs to refine the measurement of exposure dimensions in order to provide more precise targets for workplace health interventions. Third, this study treated perceived algorithmic management as a single overall construct. While theoretical and empirical justifications support this approach, we acknowledge that different dimensions of algorithmic control—such as monitoring, performance evaluation, and decision-making—may have distinct psychological effects on employees. Future research could examine these dimensions separately to uncover more nuanced mechanisms. Finally, the current model mainly focuses on the mediating pathways of health impairment and has not yet fully explored the boundary conditions in which individual characteristics (such as digital literacy and psychological resilience) and organizational environments (such as a health-supportive climate) play protective roles (21, 36). Future studies may further introduce multilevel moderating variables to systematically clarify the protective buffering resources that can effectively reduce individuals' susceptibility to the "digital burden,” thereby providing more comprehensive theoretical and empirical guidance for comprehensively building an occupational health promotion system in the era of digital intelligence.

In addition, while the modification of the WHO-5 from a 6-point to a 7-point format was methodologically justified (44, 45), the adapted version has not undergone formal metric equivalence testing against the original 6-point format. Future research should validate the 7-point version using a simultaneous data collection design to establish measurement invariance.

## Data Availability

The original contributions presented in the study are included in the article/Supplementary material, further inquiries can be directed to the corresponding author.
