# Supplementary material for: The impact of perceived algorithmic management on employees' physical and mental health: a chain mediation analysis of frustrated work autonomy and workplace AI replacement anxiety
Source: Front Public Health. 2026 Sep 1;14:1877221. doi: 10.3389/fpubh.2026.1877221 (PMC13574970; doi:10.3389/fpubh.2026.1877221)
Supplement: Supplementary file 1 [file Supplementary_file_1.docx]

**Supplementary Material**

**Supplementary Table S1. Questionnaire Items for All Scales**

*Note: All items were rated on a 7‑point Likert scale from 1 (strongly disagree) to 7 (strongly agree), except for WHO-5 items which were rated from 1 (never) to 7 (all the time).

**Perceived Algorithmic Management** (Parent-Rocheleau et al., 2024)

1.My work tasks, goals, or schedules are primarily assigned and set by algorithmic systems, rather than by human supervisors.

2.Algorithmic systems monitor and track my work performance, behavioral traces, or related data in real time.

3.Algorithmic systems automatically evaluate my work performance based on my past data or real-time performance.

4.The standardized instructions provided by algorithmic systems greatly constrain my autonomous decision-making space at work.

**Frustrated Work Autonomy** (Chen et al., 2015)

1.At work, I feel forced to do many things that I would not willingly do.

2.At work, I feel that too many things are pressing me to act.

3.My daily work feels like a series of obligations I must fulfill.

4.Most of the things I do at work feel like things I "have to do."

**Workplace AI Replacement Anxiety** (Brougham & Haar, 2018)

1.I worry that my current job will be replaced by smart technology, artificial intelligence, or algorithms (STARA) in the future.

2.I feel that due to the spread of AI and automation, my current skills will become obsolete in the future.

3.I believe that emerging technologies such as AI are fully capable of taking over my current job tasks.

4.I anticipate that the rapid development of AI will reduce my future employment opportunities.

**Employees' Physical and Mental Health (WHO-5)** (Topp et al., 2015)

1.Over the past two weeks, I have felt cheerful and relaxed.

2.Over the past two weeks, I have felt calm and peaceful.

3.Over the past two weeks, I have felt energetic and full of vitality.

4.Over the past two weeks, I have woken up feeling fresh and well-rested.

5.Over the past two weeks, my daily life has been filled with things that interest me.

**Supplementary Table S1.**

| Item | Measurement Item | Strongly Disagree | Disagree | Somewhat Disagree | Uncertain | Somewhat Agree | Agree | Strongly Agree |
| --- | --- | --- | --- | --- | --- | --- | --- | --- |
| 1 | My work tasks, goals, or schedules are primarily assigned and set by algorithmic systems, rather than by human supervisors. | 1 | 2 | 3 | 4 | 5 | 6 | 7 |
| 2 | Algorithmic systems monitor and track my work performance, behavioral traces, or related data in real time. | 1 | 2 | 3 | 4 | 5 | 6 | 7 |
| 3 | Algorithmic systems automatically evaluate my work performance based on my past data or real-time performance. | 1 | 2 | 3 | 4 | 5 | 6 | 7 |
| 4 | The standardized instructions provided by algorithmic systems greatly constrain my autonomous decision-making space at work. | 1 | 2 | 3 | 4 | 5 | 6 | 7 |
| 5 | At work, I feel forced to do many things that I would not willingly do. | 1 | 2 | 3 | 4 | 5 | 6 | 7 |
| 6 | At work, I feel that too many things are pressing me to act. | 1 | 2 | 3 | 4 | 5 | 6 | 7 |
| 7 | My daily work feels like a series of obligations I must fulfill. | 1 | 2 | 3 | 4 | 5 | 6 | 7 |
| 8 | Most of the things I do at work feel like things I "have to do." | 1 | 2 | 3 | 4 | 5 | 6 | 7 |
| 9 | I worry that my current job will be replaced by smart technology, artificial intelligence, or algorithms (STARA) in the future. | 1 | 2 | 3 | 4 | 5 | 6 | 7 |
| 10 | I feel that due to the spread of AI and automation, my current skills will become obsolete in the future. | 1 | 2 | 3 | 4 | 5 | 6 | 7 |
| 11 | I believe that emerging technologies such as AI are fully capable of taking over my current job tasks. | 1 | 2 | 3 | 4 | 5 | 6 | 7 |
| 12 | I anticipate that the rapid development of AI will reduce my future employment opportunities. | 1 | 2 | 3 | 4 | 5 | 6 | 7 |
| 13 | Over the past two weeks, I have felt cheerful and relaxed. | 1 | 2 | 3 | 4 | 5 | 6 | 7 |
| 14 | Over the past two weeks, I have felt calm and peaceful. | 1 | 2 | 3 | 4 | 5 | 6 | 7 |
| 15 | Over the past two weeks, I have felt energetic and full of vitality. | 1 | 2 | 3 | 4 | 5 | 6 | 7 |
| 16 | Over the past two weeks, I have woken up feeling fresh and well-rested. | 1 | 2 | 3 | 4 | 5 | 6 | 7 |
| 17 | Over the past two weeks, my daily life has been filled with things that interest me. | 1 | 2 | 3 | 4 | 5 | 6 | 7 |

Note: Perceived Algorithmic Management (Items 1–4); Frustrated Work Autonomy (Items 5–8); Workplace AI Replacement Anxiety (Items 9–12); Employees’ Physical and Mental Health (Items 13–17).
