# Supplementary material for: The impact of perceived algorithmic management on employees' physical and mental health: a chain mediation analysis of frustrated work autonomy and workplace AI replacement anxiety
Source: Front Public Health. 2026 Sep 1;14:1877221. doi: 10.3389/fpubh.2026.1877221 (PMC13574970; doi:10.3389/fpubh.2026.1877221)
Supplement: Supplementary file 2 [file Supplementary_file_2.docx]

**Survey Questionnaire**

**Part 1: Demographic Information**

1.Gender: A. Male B. Female

2.Age: A. 25 and below B. 26–35 C. 36–45 D. 46–55 E. 56 and above

3.Highest education level: A. Junior college or below B. Bachelor's degree C. Master's degree D. Doctorate or above

4.Enterprise type: A. State-owned enterprise B. Private enterprise C. Foreign-funded/Joint venture D. Public institution/Government agency E. Other

5.Region where your enterprise is located: A. Eastern Region B. Central Region C. Western Region D. Northeast Region

(Eastern Region: Beijing, Tianjin, Hebei, Shanghai, Jiangsu, Zhejiang, Fujian, Shandong, Guangdong, and Hainan; Central Region: Shanxi, Anhui, Jiangxi, Henan, Hubei, and Hunan; Western Region: Inner Mongolia, Guangxi, Chongqing, Sichuan, Guizhou, Yunnan, Tibet, Shaanxi, Gansu, Qinghai, Ningxia, and Xinjiang; Northeast Region: Liaoning, Jilin, and Heilongjiang)

6.Total years of work experience: A. Less than 1 year B. 1–3 years (inclusive) C. 4–8 years (inclusive) D. 9–15 years (inclusive) E. Over 15 years

7.Current job level: A. Frontline employee (no subordinates) B. Frontline manager (e.g., supervisor, team leader) C. Middle manager (e.g., department manager) D. Senior manager (e.g., director, vice president or above)

8.Primary job nature: A. R&D/Technical B. Production/Manufacturing/Logistics C. Marketing/Sales/Customer Service D. HR/Administration/Finance and other functional roles E. Other

| Item | Measurement Item | Strongly Disagree | Disagree | Somewhat Disagree | Uncertain | Somewhat Agree | Agree | Strongly Agree |
| --- | --- | --- | --- | --- | --- | --- | --- | --- |
| 1 | My work tasks, goals, or schedules are primarily assigned and set by algorithmic systems, rather than by human supervisors. | 1 | 2 | 3 | 4 | 5 | 6 | 7 |
| 2 | Algorithmic systems monitor and track my work performance, behavioral traces, or related data in real time. | 1 | 2 | 3 | 4 | 5 | 6 | 7 |
| 3 | Algorithmic systems automatically evaluate my work performance based on my past data or real-time performance. | 1 | 2 | 3 | 4 | 5 | 6 | 7 |
| 4 | The standardized instructions provided by algorithmic systems greatly constrain my autonomous decision-making space at work. | 1 | 2 | 3 | 4 | 5 | 6 | 7 |
| 5 | At work, I feel forced to do many things that I would not willingly do. | 1 | 2 | 3 | 4 | 5 | 6 | 7 |
| 6 | At work, I feel that too many things are pressing me to act. | 1 | 2 | 3 | 4 | 5 | 6 | 7 |
| 7 | My daily work feels like a series of obligations I must fulfill. | 1 | 2 | 3 | 4 | 5 | 6 | 7 |
| 8 | Most of the things I do at work feel like things I "have to do." | 1 | 2 | 3 | 4 | 5 | 6 | 7 |
| 9 | I worry that my current job will be replaced by smart technology, artificial intelligence, or algorithms (STARA) in the future. | 1 | 2 | 3 | 4 | 5 | 6 | 7 |
| 10 | I feel that due to the spread of AI and automation, my current skills will become obsolete in the future. | 1 | 2 | 3 | 4 | 5 | 6 | 7 |
| 11 | I believe that emerging technologies such as AI are fully capable of taking over my current job tasks. | 1 | 2 | 3 | 4 | 5 | 6 | 7 |
| 12 | I anticipate that the rapid development of AI will reduce my future employment opportunities. | 1 | 2 | 3 | 4 | 5 | 6 | 7 |
| 13 | Over the past two weeks, I have felt cheerful and relaxed. | 1 | 2 | 3 | 4 | 5 | 6 | 7 |
| 14 | Over the past two weeks, I have felt calm and peaceful. | 1 | 2 | 3 | 4 | 5 | 6 | 7 |
| 15 | Over the past two weeks, I have felt energetic and full of vitality. | 1 | 2 | 3 | 4 | 5 | 6 | 7 |
| 16 | Over the past two weeks, I have woken up feeling fresh and well-rested. | 1 | 2 | 3 | 4 | 5 | 6 | 7 |
| 17 | Over the past two weeks, my daily life has been filled with things that interest me. | 1 | 2 | 3 | 4 | 5 | 6 | 7 |

Note: Perceived Algorithmic Management (Items 1–4); Frustrated Work Autonomy (Items 5–8); Workplace AI Replacement Anxiety (Items 9–12); Employees‘ Physical and Mental Health (Items 13–17).
